# Ultrasound-guided percutaneous nephrolithotomy without fluoroscopy: early surgical experience with video-based technical insights

**DOI:** 10.3389/fsurg.2025.1706729

**Published:** 2025-10-30

**Authors:** Chenghao Tan, Xin Li, Xiaochun Zhang, Gang Wang, Gengyan Xiong

**Affiliations:** Department of Urology, Peking University First Hospital, Institute of Urology, Peking University, National Urological Cancer Center, Beijing, China

**Keywords:** ultrasound-guided, percutaneous nephrolithotomy, radiation-free, video-based, technical insights

## Abstract

**Objectives:**

To assess the feasibility, safety, and short-term outcomes of ultrasound-guided percutaneous nephrolithotomy (PCNL) performed by a single early-career urologist without fluoroscopic guidance.

**Methods:**

We retrospectively analyzed 70 consecutive ultrasound-guided PCNL cases performed independently by a single early-career urologist at Peking University First Hospital. All procedures employed single-tract, rigid ureteroscopy-assisted access under real-time ultrasound guidance without fluoroscopy. Perioperative parameters included access success, operative time, hemoglobin drop, stone-free rate, and complications. An accompanying educational video was included to facilitate reproducibility.

**Results:**

The stone-free rate after a single procedure was 75.7%, rising to 88.6% at 6-month follow-up. No major complications occurred. The mean stone size was 2.22 cm. Mean access time was 9 min. Access was successfully achieved in all cases. These outcomes were achieved despite the surgeon's early learning curve, reflecting a high degree of technical feasibility and safety under structured mentorship.

**Conclusions:**

Ultrasound-guided PCNL is a safe, effective, and radiation-free alternative to fluoroscopy-guided procedures. It can be reliably adopted early in surgical practice. The accompanying video supports broader understanding and dissemination of this radiation-free approach, especially in resource-limited or high-volume centers.

## 1 Introduction

Ultrasound-guided percutaneous nephrolithotomy (PCNL) has emerged as a radiation-free, cost-effective technique with high success rates when performed by trained urologists (1, 2). It is widely adopted in China, where it has become a standard approach (3). Compared to fluoroscopic guidance, ultrasound offers multiple advantages, including reduced radiation exposure for both patients and surgeons, lower procedural costs (4), real-time visualization of renal structures, detection of radiolucent stones, improved delineation of adjacent organs, and enhanced identification of vascular anatomy via Doppler imaging (5, 6).

Despite its advantages, ultrasound-guided PCNL has not replaced fluoroscopy in many regions (7). C-arm systems remain dominant because they provide an easily interpretable two-dimensional view that is familiar to most surgeons, are embedded in long-standing training curricula, and appear technically simpler during access establishment. However, fluoroscopy is bulky, expensive, and associated with cumulative radiation exposure. Moreover, its reliance on ionizing radiation necessitates additional safety precautions for both patients and operating personnel.

In resource-limited settings, ultrasound-guided PCNL is particularly valuable. Many regions, such as Sub-Saharan Africa, lack access to advanced endourological equipment (8), yet face a significant burden of nephrolithiasis. Obstructive stones leading to septic shock require urgent decompression, and ultrasound facilitates rapid percutaneous nephrostomy placement, stabilizing critically ill patients when fluoroscopic guidance is unavailable. Beyond emergency scenarios, ultrasound-guided PCNL remains the preferred modality for complex stone disease, especially in cases involving hydronephrosis, infection, or anatomical abnormalities. However, it is contraindicated in patients with severe renal dysfunction, coagulopathy, uncontrolled infections, high anesthetic risk, specific anatomical anomalies, or pregnancy.

Nevertheless, the global adoption of ultrasound-guided PCNL remains limited by its perceived technical difficulty and learning curve. Previous publications have mainly relied on textual descriptions or static images (9, 10), which may not adequately convey the real-time spatial orientation required for safe renal access. Evidence from recent learning-curve analyses indicates that novice surgeons can achieve proficiency with structured mentorship and repeated practice (11, 12), yet standardized visual training materials are still scarce. To address this gap, we present a concise, step-by-step methodology supplemented by a high-quality instructional video. This approach aims to enhance procedural understanding, support early surgical proficiency, and promote the wider adoption of this radiation-free technique during the initial phase of independent practice.

## 2 Materials and equipment

Preoperative preparation included computed tomography for evaluation of renal and perirenal anatomy, stone burden, and anatomical variations, as well as Doppler ultrasound, which also served as the primary intraoperative guidance tool. Urine culture and sensitivity testing were performed to guide prophylactic antibiotics, typically broad-spectrum cephalosporins or fluoroquinolones, administered according to institutional protocols. Normal saline was used as the irrigation solution.

Percutaneous access was obtained using an echogenic 18–24G puncture needle and J-tipped guidewire (Urovision, Germany), followed by sequential fascial dilators (8F–18F, Urovision) or balloon dilation with an access sheath. Endoscopic equipment consisted of a rigid ureteroscope (F8/9.8, Richard Wolf GmbH, Germany) and a nephroscope for tract confirmation, lithotripsy, and stone retrieval, with a flexible nephroscope selectively used to inspect residual fragments. Stone fragmentation was performed using a Lumenis Pulse™ holmium:YAG laser system (Boston Scientific, USA) with an energy of 1.8 J and frequency of 25 Hz.

Drainage was secured by a double-J ureteral stent [Becton Dickinson (BD), USA] tailored to patient anatomy, a premeasured ureteral catheter (25–30 cm), and a nephrostomy tube (F14–18) when clinically indicated. Standard anesthesia equipment, monitoring devices, sterile drapes, ultrasound gel, and hemostatic materials were routinely prepared.

## 3 Methods

### 3.1 Study design and patients

This is a retrospective single-surgeon analysis of the first 70 consecutive ultrasound-guided PCNL cases, conducted within an institution (Peking University First Hospital, PUFH) that has accumulated over 3,000 PCNL procedures since 2000. Inclusion criteria: adult patients who underwent single-tract, ultrasound-guided PCNL performed independently by a single urologist at PUFH, with complete perioperative and follow-up data. Exlusion criteria: patients with complex or staghorn calculi, solitary kidney, active urinary tract infection, uncorrected coagulopathy, or contraindication to general anesthesia. These were the first 70 cases in which Dr. Xiong G. independently performed this technique. We retrospectively collected and analyzed preoperative baseline characteristics, intraoperative parameters, and postoperative outcomes (follow-up results) from the institutional electronic medical record system and operative video archives.

To further illustrate the progress, we selected a typical patient (who provided full informed consent) hospitalized at PUFH to record this surgical procedure (Supplementary Video 1). This surgery was also performed by the same surgeon (Xiong G.), who has subsequently attained a high level of expertise in this technique.

### 3.2 Surgical technique

#### 3.2.1 Preoperative puncture planning

**Figure 1:**
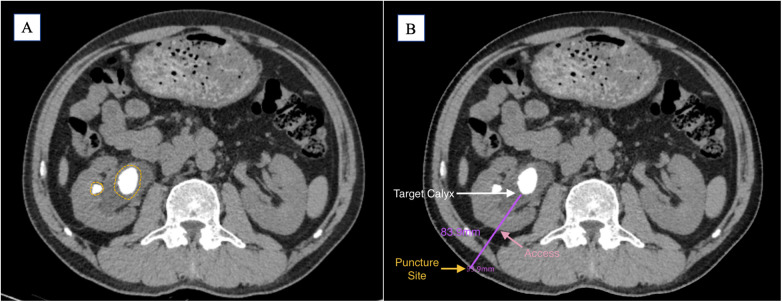
**(A)** Preoperative CT imaging shows the main stone located in the renal pelvis, with a small stone visible in the mid-anterior calyx (yellow circle). **(B)** Based on the preoperative findings, the target calyx is selected (white arrow); the shortest perpendicular path to the skin is indicated (pink arrow); the puncture point is marked (yellow arrow); the estimated puncture tract length is 83.9 mm (purple line).

The puncture trajectory should target the central axis of the target calyx, adhering to the shortest and safest path through the renal cortex. While a perpendicular skin entry is ideal, angulation may be required to optimize precision and minimize tissue traversal (Figure 1).

#### 3.2.2 Ureteral catheter placement

Under general anesthesia, position the patient in lithotomy. A premeasured ureteral catheter (25–30 cm, based on preoperative CT) is inserted via cystoscopy. Correct positioning is confirmed by continuous, clear urinary efflux from the catheter. In patients without hydronephrosis, the collecting system was gently distended by connecting the ureteral catheter to a saline infusion bottle and adjusting its height to control hydrostatic pressure, thereby achieving a moderate and safe level of artificial hydronephrosis. This method allows real-time adjustment of intrapelvic pressure to optimize ultrasound visualization while minimizing the risk of overdistension.

#### 3.2.3 Ultrasound-guided percutaneous access

***Key Technical Steps:***

1. **Calyceal Entry Selection:**

All patients were positioned in the prone position before the procedure began. The needle was then advanced under continuous ultrasound guidance until the echogenic tip entered the target calyx, and aspiration of clear urine confirmed successful intraluminal placement.

1. **Guidewire Insertion and Safety Assessment:**

Insert a J-tipped guidewire through the needle. Gently advance/retract the wire to evaluate the critical distance between the wire tip and renal pelvis wall, ensuring a safe margin for dilation.

1. **Dilation Protocol:**

Initiate tract dilation with an F8 dilator, guided by the blood trace length (post-needle withdrawal) and preoperative safety margins. Monitor for urine efflux between the guidewire and dilator, confirming entry into the collecting system. Progressively upsize dilators while maintaining guidewire stability.

Principle: “Shallow access is safer than deep.” Excessive depth risks pelvic perforation, while insufficient depth permits controlled re-dilation.

#### 3.2.4 Access tract confirmation and scope insertion

After tract establishment, insert an F8/9.8 ureteroscope through the sheath. Visual confirmation of renal stones confirms successful access. Measure the exact depth and adjust dilation as needed.

#### 3.2.5 Intraoperative lithotripsy

It is generally recommended to evaluate for residual stones using ultrasound. In this case, however, the presence of concomitant ureteral calculi necessitates the use of a flexible ureteroscope, which also enables direct inspection for residual fragments. Stones should be fragmented systematically under direct vision, with an emphasis on achieving single-session clearance. A flexible ureteroscopic examination from the renal pelvis to the bladder should be performed to rule out any remaining stone fragments.

#### 3.2.6 Post-procedural stent and catheter placement

1. **Double-J Stent Positioning:**

Advance the stent into the renal pelvis using the premeasured dilation depth as a guide. Confirm proximal placement endoscopically; reposition if migration occurs.

1. **Nephrostomy Tube Insertion:**

Place under ultrasound guidance, referencing the puncture depth. Validate patency via continuous fluid drainage from the external port. A nephrostomy tube was placed in cases of bleeding risk, infection, or expected re-entry. Tubeless PCNL was performed when hemostasis was satisfactory, no significant residual fragments or infection were present, and tract stability was confirmed.

1. **Final Confirmation:**

Perform bladder ultrasonography to visualize the distal stent. Confirm complete stone clearance and stent position via postoperative KUB radiography when necessary.

### 3.3 Technical nuances for success

Ultrasound Optimization: Maintain needle tip visibility by minimizing hand tremor and adjusting probe angulation.

Guidewire Integrity: Avoid straightening the J-tip during manipulation to prevent false depth assessments.

Tract Establishment: Prioritize depth control during dilation, using the first appearance of blood as a reference point under the principle of “Shallow access is safer than deep.” Continuously adjust the guidewire or hook wire to dynamically assess tract depth. Final confirmation of successful access should be performed under direct vision with flexible ureteroscopy.

## 4 Results

We analyzed the initial 70 cases of ultrasound-guided PCNL independently performed by Dr. Gengyan Xiong, a urologist in early independent practice at our center. All procedures were performed using a single percutaneous tract without the use of flexible nephroscopy. The mean maximal stone diameter was 2.22 cm. The average operative time was 73.9 min, with a mean access time of 9 min. In 49 patients, the puncture site was located in the middle calyx, and in 43 cases, the access tract was established above the 12th rib. Postoperatively, the mean hemoglobin drop on day 1 was 10.2 g/L, and the average hospital stay was 4.7 days. All perioperative complications were assessed and classified according to the Clavien–Dindo grading system, with none exceeding Grade I in this series. The stone-free rate (no residual fragment >2 mm on non-contrast low-dose CT or ultrasound postoperatively) after a single-stage procedure was 75.7% (53 of 70). Furthermore, after a median follow-up of 50 months (range, 18–58 months), we found that most patients had already achieved a stone-free rate of 88.6% (63 of 70) by 6 months postoperatively, and none reported any discomfort or symptomatic episodes requiring a secondary procedure for stone clearance during the follow-up period as shown in Table 1.

**Table 1:** Clinical outcomes of single-channel, rigid ureteroscopy-assisted ultrasound-guided percutaneous nephrolithotomy (PCNL) in the initial 70 cases performed by Dr. Gengyan Xiong.

The patients’ demographic and preoperative clinical characteristics		
Age, years, mean (SD)		52.5 (11.2)
BMI, kg/m^2^, mean (SD)		25.8 (3.58)
Sex—male/female, *n*		50/20
Laterality—left/right, *n*		40/29
Stone max diameter, cm, mean (SD)		2.22 (1.20)
Depth of hydronephrosis, cm, mean (SD)		1.39 (1.37)
Preoperative serum creatinine level, umol/L, mean (SD)		84.8 (19.5)
Comorbidities, *n* (%) Hypertension/Diabetes mellitus		28 (40)/15 (21.4)
Operative characteristics and follow-up results		
Operating time, min, mean (SD)		73.9 (27.8)
Access time, min, mean (SD)		9.0 (4.6)
Puncture site	Upper calyx	5
	Middle calyx	49
	Lower calyx	16
Location of access tract	Above 11th rib	0
	Above 12th rib	43
	Below 12th rib	13
Postoperative serum, creatinine level, umol/L, mean (SD)		67.2 (20.8)
Haemoglobin drop, g/L, mean (SD)		10.2 (7.5)
Hospital stay, days, mean (SD)		4.7 (1.1)
Stone free after one stage operation, *n* (%)		53 (75.7)
Stone free after 6 months, *n* (%)		63 (88.6)
Postoperative complications, CD grade, *n* (%)		0 (0)
Follow-up time (mo), median (range)		50 (18–58)

CD, Clavien–Dindo; SD, standard deviation.

Data are presented as *n* (%), mean (SD) or median (range).

## 5 Discussion

This study highlights the safety, feasibility, and reproducibility of ultrasound-guided PCNL in real-world clinical practice. Based on the early independent experience of a newly practicing urologist, the technique achieved a favorable stone-free rate, minimal hemoglobin loss, and no major access-related complications. These results demonstrate that, when performed within a structured learning environment, ultrasound-guided PCNL can be safely adopted even during the early phase of surgical practice, reflecting the maturity of the approach in experienced centers and its potential for broader dissemination.

Our findings align with prior literature on the learning curve of ultrasound-guided PCNL. Xiao et al. recently reported that operative parameters stabilized after approximately 40 cases in a cohort of complex renal stones, underscoring that technical proficiency can be achieved within a moderate learning curve (11). Similarly, a 2025 systematic review by Wirtzfeld et al. found that puncture success rates approach 100% after 25–50 cases, with operative efficiency improving significantly beyond 36 cases (12). These data support our observation that stable and reproducible outcomes can be achieved by early-career surgeons under structured mentorship.

Compared with conventional fluoroscopy-guided PCNL, ultrasound guidance presents unique challenges in spatial orientation, especially during puncture and guidewire manipulation—steps that are not continuously visualized. These difficulties have been cited as major barriers to adoption in Western urologic practice. Our results confirm these challenges but also demonstrate that they can be effectively mitigated through standardized protocols, emphasis on tactile and visual cues, and real-time supervision. Importantly, our center has performed PCNL exclusively under ultrasound guidance since 2011, illustrating that complete transition from fluoroscopy is both feasible and sustainable when institutional support and training infrastructure are available.

The clinical implications of this work are twofold. First, ultrasound-guided PCNL provides a viable radiation-free alternative in high-income settings, where fluoroscopy remains the standard despite growing concerns regarding cumulative radiation exposure for both patients and operating staff. Second, in resource-limited regions where fluoroscopic equipment may not be available, ultrasound guidance offers a pragmatic and potentially life-saving solution for obstructive uropathy. Tract creation can be safely achieved under local anesthesia, making the procedure accessible even in environments with limited anesthetic resources—particularly valuable in emergent situations such as septic shock due to upper urinary tract obstruction. By eliminating dependence on x-ray imaging and general anesthesia, the technique significantly broadens access to definitive surgical care.

We also emphasize the educational value of the accompanying operative video. Unlike previous reports that rely primarily on static images or textual descriptions, the present video demonstrates real-time ultrasound imaging, puncture trajectory alignment, and guidewire placement, providing dynamic visual feedback that can shorten the learning curve for new adopters. Such multimedia resources may play a pivotal role in bridging the gap between theoretical knowledge and practical execution, thereby promoting global adoption.

This study has several limitations. It is a single-center, retrospective analysis based on one surgeon's early experience under institutional mentorship, which may limit generalizability. Selection bias is possible, as early cases were carefully chosen to ensure safety during the learning phase. Stone complexity was not stratified using formal nephrolithometric systems such as the Guy's Stone Score (GSS). Given the limited sample size and the focus on an early single-surgeon cohort, subgroup analysis by stone complexity would likely have been underpowered and not statistically meaningful. Future multicenter studies with larger and more heterogeneous populations may further clarify the impact of stone complexity on outcomes. Although previous studies have demonstrated that ultrasound-guided PCNL is feasible and safe in obese patients with appropriate technical adjustments and depth control (13, 14), the present cohort included relatively few obese individuals. This reflects both the limited number of high-BMI patients undergoing surgery at our center and the higher technical demands of the ultrasound-guided approach during the early learning phase, which may have influenced patient selection. Additionally, although our educational video aims to capture procedural nuances, hands-on experience and direct supervision remain essential for skill transfer. For institutions transitioning to ultrasound guidance, hybrid strategies—such as combining ultrasound with limited fluoroscopic assistance or using flexible ureteroscopic placement for visual puncture confirmation—may serve as effective transitional models to ensure patient safety.

Future perspectives include the development of standardized training curricula and multicenter implementation frameworks. Prospective trials comparing ultrasound- and fluoroscopy-guided PCNL across diverse clinical environments are warranted to define the optimal approach under varying resource conditions. We hope that the instructional materials and methodological standardization introduced in this study will contribute to structured, global dissemination of ultrasound-guided PCNL as a safe, reproducible, and radiation-free alternative for kidney stone surgery.

## Data Availability

The raw data supporting the conclusions of this article will be made available by the authors, without undue reservation.
